# Full-Length Genomic Analysis of Korean Porcine Sapelovirus Strains

**DOI:** 10.1371/journal.pone.0107860

**Published:** 2014-09-17

**Authors:** Kyu-Yeol Son, Deok-Song Kim, Joseph Kwon, Jong-Soon Choi, Mun-Il Kang, Graham J. Belsham, Kyoung-Oh Cho

**Affiliations:** 1 Laboratory of Veterinary Pathology, College of Veterinary Medicine, Chonnam National University, Gwangju, Republic of Korea; 2 Division of Life Science, Korea Basic Science Institute, Yuseong-gu, Daejeon, Republic of Korea; 3 National Veterinary Institute, Technical University of Denmark, Kalvehave, Denmark; The University of Hong Kong, Hong Kong

## Abstract

Porcine sapelovirus (PSV), a species of the genus *Sapelovirus* within the family *Picornaviridae*, is associated with diarrhea, pneumonia, severe neurological disorders, and reproductive failure in pigs. However, the structural features of the complete PSV genome remain largely unknown. To analyze the structural features of PSV genomes, the full-length nucleotide sequences of three Korean PSV strains were determined and analyzed using bioinformatic techniques in comparison with other known PSV strains. The Korean PSV genomes ranged from 7,542 to 7,566 nucleotides excluding the 3′ poly(A) tail, and showed the typical picornavirus genome organization; 5′untranslated region (UTR)-L-VP4-VP2-VP3-VP1-2A-2B-2C-3A-3B-3C-3D-3′UTR. Three distinct *cis*-active RNA elements, the internal ribosome entry site (IRES) in the 5′UTR, a *cis*-replication element (*CRE*) in the 2C coding region and 3′UTR were identified and their structures were predicted. Interestingly, the structural features of the *CRE* and 3′UTR were different between PSV strains. The availability of these first complete genome sequences for PSV strains will facilitate future investigations of the molecular pathogenesis and evolutionary characteristics of PSV.

## Introduction

Picornaviruses are a family of positive-sense single stranded RNA viruses within the order Picornavirales [1]. They can cause intestinal, respiratory, neurological, cardiac, hepatic, mucocutaneous, and systemic diseases of varying severity in humans and animals [2]. Although different picornaviruses show various degrees of relatedness, all picornaviruses share a similar genomic organization, which consists of a covalently linked 5′ terminal protein called VPg (Viral Protein genome-linked), a 5′ untranslated region (UTR), a large open reading frame (ORF), a 3′ UTR and a poly(A) tail of variable length [2], [3]. The genomic RNA of picornaviruses harbor several distinct *cis*-active RNA elements which are required for viral RNA replication; an internal ribosome entry site (IRES) in the 5′UTR, a *cis*-replication element (*CRE*) within the ORF [3]–[6] or the 5′ UTR [7], the 3′ UTR, and the 3′ poly(A) tail [3]. Currently, five different types of IRES element [8] that direct cap-independent translation initiation on the viral RNA to produce the polyprotein have been identified from the primary sequence, secondary structure, location of the initiation codon and activity in different cell types [9], [10]. In most picornaviruses, the polyprotein encoded by the ORF is cleaved into four structural viral particle proteins (VP4, VP2, VP3 and VP1) and seven non-structural proteins (2A, 2B, 2C, 3A, 3B, 3C and 3D). In addition, the members of the genera *Cardiovirus*, *Aphthovirus*, *Erbovirus*, *Kobuvirus*, *Teschovirus, Senecavirus* and *Sapelovirus* possess a leader (L) protein at the N-terminus of polyprotein [11].

Although simian type 2 picornaviruses (SV-2-like viruses) and porcine enterovirus 8 (PEV-8) were once classified in the genus *Enterovirus*, SV-2-like viruses and PEV-8 along with duck picornavirus TW90A have an L protein at the N-terminus of the polyprotein that is lacking in the enteroviruses [12]–[14]. Moreover, those viruses contain distinct 2A proteins from those of the *Enterovirus* genus, and a highly divergent 5′UTR with a type IV IRES [12], [14]–[16]. Due to these particular genetic properties, these simian, avian, and porcine picornaviruses are now assigned as members of a new picornavirus genus, *Sapelovirus* [12]–[15], [17], [18].

PSV infections have been associated with a wide spectrum of symptoms ranging from asymptomatic infection to clinical signs including diarrhea, pneumonia, polioencephalomyelitis, and reproductive disorders [19]–[20]. Although PSVs can be important pathogens because of their wide distribution and high prevalence [21]–[25], the near-complete genomic sequences of only three PSV strains have been reported previously; one from the U.K. (V13 strain) and two from China (csh and YC2011 strains). This prompted us to characterize the full-length genetic properties of Korean PSV strains in comparison with those of other known PSV strains.

During an epidemiological study on PSV infections in the fecal samples of piglets with diarrhea in South Korea, three PSV strains were isolated. These Korean PSV strains were characterized using an immunofluorescence assay (IFA) with a monoclonal antibody specific for a PSV capsid protein, RT-PCR assay with primer pair specific for the PSV VP1 coding region and transmission electron microscopy. Furthermore, bioinformatic techniques were employed to analyze the complete viral genomes of the three newly isolated strains in comparison with the other known PSV strains.

## Materials and Methods

### Origin of virus strains

The diarrheic fecal samples, collected from piglets in 45 different herds in South Korea during 2004–2007, were screened for PSV infections using RT-PCR and nested-PCR assays with primer pairs specific for the PSV VP1 coding region [26]. Among the PSV-positive fecal samples, three fecal samples which were strongly positive for PSV by RT-PCR were used to isolate PSVs using a porcine kidney cell line [22], LLC-PK. In brief, the RT-PCR positive fecal samples were diluted 10-times with 0.01 M phosphate-buffered saline (PBS, pH 7), vortexed for 30 s and centrifuged at 1200×g for 20 min. The supernatants were filtered through 0.2-µm syringe filters. Filtered supernatants were serially diluted 10-times with Eagle’s minimal essential medium (EMEM) containing 1% antibiotics (Penicillin, Streptomycin, and Amphotericin B) and 1% NaHCO_3_, and used to infect cells in 6-well plates. The suspensions were absorbed for 1 h with occasional rocking, and EMEM containing 1% antibiotics and 1% NaHCO_3_ was added. The cultures were incubated for 3 to 4 days at 37°C in a 5% CO_2_ atmosphere and examined daily for cytopathic effects (CPE). Isolated PSVs were cloned by triple plaque purification. The PSV strains (KS04105, KS05151 and KS055217) were passaged eight times in LLC-PK cells, including isolation, adaptation, and triple plaque purification. The isolated viruses were confirmed as PSVs by an IFA, RT-PCR and transmission electron microscopy (TEM) assays, as described below.

### Transmission electron microscopy (TEM)

LLC-PK cells infected with each of the above strains and showing over 70% CPE were frozen and thawed thrice, and centrifuged at 2,000×g for 30 min. To obtain purified virus, each supernatant was ultra-centrifuged at 200,000×g for 5 h at 4°C in a S58A-0015 rotor (Hitachi, Tokyo, Japan). The resulting pellets were resuspended in 40 µl of water and mixed with an equal volume of 2% (w/v) sodium phosphotungstic acid at pH 7.0. The samples were placed onto a formvar grid (Electron Microscopy Sciences, Hatfield, USA) for 5 min, and then excess liquid was removed by filter paper. The samples were examined using a High Resolution Transmission Electron Microscope (Hitachi) for the determination of purity of virus stock at Gwangju Center of Korea Basic Science Institute.

### Immunofluorescence assay (IFA)

To characterize the PSV strains, the IFA was performed with a 153/5B5 (IgG2a) monoclonal antibody specific for the PSV capsid protein (kindly provided by Dr. M Dauber, Friedrich-Loeffler-Institut, Greifswald-Insel Riems, Germany). Briefly, LLC-PK cells were infected with each strain, incubated for 18 h as above, fixed in 80% acetone for 5 min at 4°C, and allowed to air dry. Slides were washed thrice with PBS (pH 7.2), and incubated overnight at 4°C using a 1∶40 dilution of monoclonal antibody specific for PSV capsid protein diluted in PBS (pH 7.2). Slides were washed thrice with PBS (pH 7.2), and incubated with FITC-conjugated goat anti-mouse IgG antibody (Santa Cruz biotechnology, Santa Cruz, USA) diluted 1∶100 in PBS (pH 7.2) for 1 h at room temperature. After washing twice with PBS (pH 7.2), slides were stained with 4′,6-diamidino-2-phenylindole (DAPI) (Invitrogen, Lohne, Germany), and examined using a LSM confocal scanning microscope (Carl Zeiss, Jena, Germany).

### RNA extraction and RT-PCR

Total RNA was extracted from the lysates of LLC-PK cells infected with each strain using the AccuPrep Viral RNA extraction kit (Bioneer, Daejeon, Korea) according to the manufacturer’s instructions. To detect and amplify PSV RNA, RT-PCR with a primer pair specific for the PSV VP1 coding sequence (Table S1 in File S1) was performed. To characterize the complete full-length genome sequences of each strain, ten primer sets (Table S1 in File S1) were designed to amplify the complete ORF sequences of each PSV strain based on the published genomic sequences of the PSV-V13 (GenBank ID: NC_003987), csh (GenBank ID: HQ875059) and YC2011 strains (GenBank ID: JX286666). Standard one-step RT-PCR assays were performed as previously described [25].

### 5′ and 3′ cDNA syntheses

cDNA of each strain was synthesized by the SMARTer Rapid Amplification of cDNA Ends (RACE) cDNA amplification kit (Clontech, Mountain View, USA) according to the manufacturer’s instructions. For generating 3′ RACE-ready cDNA, 3.75 µl of the poly(A) tailed RNA and 1 µl of 3′-cDNA Synthesis (CDS) Primer A were mixed and heated to 72°C for 3 min, followed by cooling to 42°C for 2 min using a thermo cycler. For generating 5′ RACE-ready cDNA, 3.75 µl of total RNA was mixed with 1 µl of 5′-CDS primer A, incubated at 72°C for 3 min, and cooled at 22°C for 2 min. The denatured RNA for each 3′ and 5′ cDNA generation was mixed with a reaction mixture composed of 2 µl 5×First-Strand Buffer, 1 µl dithiothreitol (DTT) (20 mM), 1 µl dNTP mix (10 mM), 0.25 µl RNase inhibitor (40 U/µl), and 1 µl SMARTScriber Reverse Transcriptase (100 U). Samples were incubated at 42°C for 90 min and heated at 70°C for 10 min. The synthesized cDNAs were diluted with 7 µl of Tricine-EDTA buffer and used for RACE PCR.

### RACE PCR, cloning and sequencing

For the generation of 3′ and 5′ RACE PCR reactions, Advantage 2 Polymerase Mix (Clontech) was used; 5 µl of 3′/5′-RACE-Ready cDNA, 32 µl of PCR-grade water, 5 µl of 10×Advantage 2 PCR Buffer, 1 µl dNTP Mix (10 mM), 5 µl Universal Primer Mix (10 X), 1 µl of 50 pmol/ml gene-specific primer (GSP) for 3′ and 5′ RACE (Table S1 in File S1), and 1 µl of 50×Advantage 2 Polymerase Mix. The reaction was performed with the following thermal cycling program: 5 cycles of 94°C or 94.5°C for 30 sec and 72°C for 2 min or 3 min; 5 three-step cycles of 94°C or 94.5°C for 30 sec, 65°C (applied melting temperature of GSP) for 30 sec, and 72°C for 2 min or 3 min; 25 three-step cycles of 94°C or 94.5°C for 30 sec, 60°C (applied lowered Tm values of GSP by 3°C to 5°C) for 30 sec, and 72°C for 2 min or 3 min.

The RACE PCR products were separated on a 1.5% (m/v) agarose gel; the bands were excised and purified using a Purelink Quick Gel Extraction kit (Invitrogen). The products were ligated into TA Vector Systems (Enzynomics, Daejeon, Korea) and introduced into DH5α competent cells. Individual colonies were grown and plasmid was purified using Hybrid-Q Plasmid (GeneAll, Seoul, Korea). Sequencing was performed using an ABI System 3700 automated DNA sequencer (Applied Biosystems, Foster, USA).

### Full-length genomic characterization and secondary structure prediction

To generate complete nucleotide sequences for each PSV strain, both 5′ and 3′ end sequences of each strain were assembled with the nucleotide sequences of the internal ORF sequences. The complete full-length genomic and individual protein coding sequences of three PSV strains were compared with those of the other known PSV strains (Table S2 in File S1) using the DNA Basic module (DNAsis MAX, Alameda, USA). Phylogenetic analyses based on nucleotide and amino acid alignments were performed using the neighbor-joining method with 1000 bootstrap replicates and UPGMA Molecular Evolutionary Genetics Analysis (MEGA version 5.2) employing pair-wise distance comparisons [27]. Sequence identity calculations for the three PSV strains with those of the other known PSV strains were performed using the homology and distance matrices method of DNAMAN version 6.0 program (Lynnon, Vaudreuil, Canada). Secondary structure elements in the PSV genomes were modeled using CLC Main Workbench version 6.8.2 program (CLC bio, Katrinebjerg, Denmark).

### Ethics statement

No specific approval was needed since the fecal samples were voluntarily submitted by the farms for pathogen screening in our laboratory. No other specific permits were required for the described field studies. The locations where we sampled are not protected in any way. The field studies did not involve endangered or protected animal species. Before beginning work on the study, we contacted the farm owners and obtained their permission.

## Results

### Virus isolation and identification

**Figure 1:**
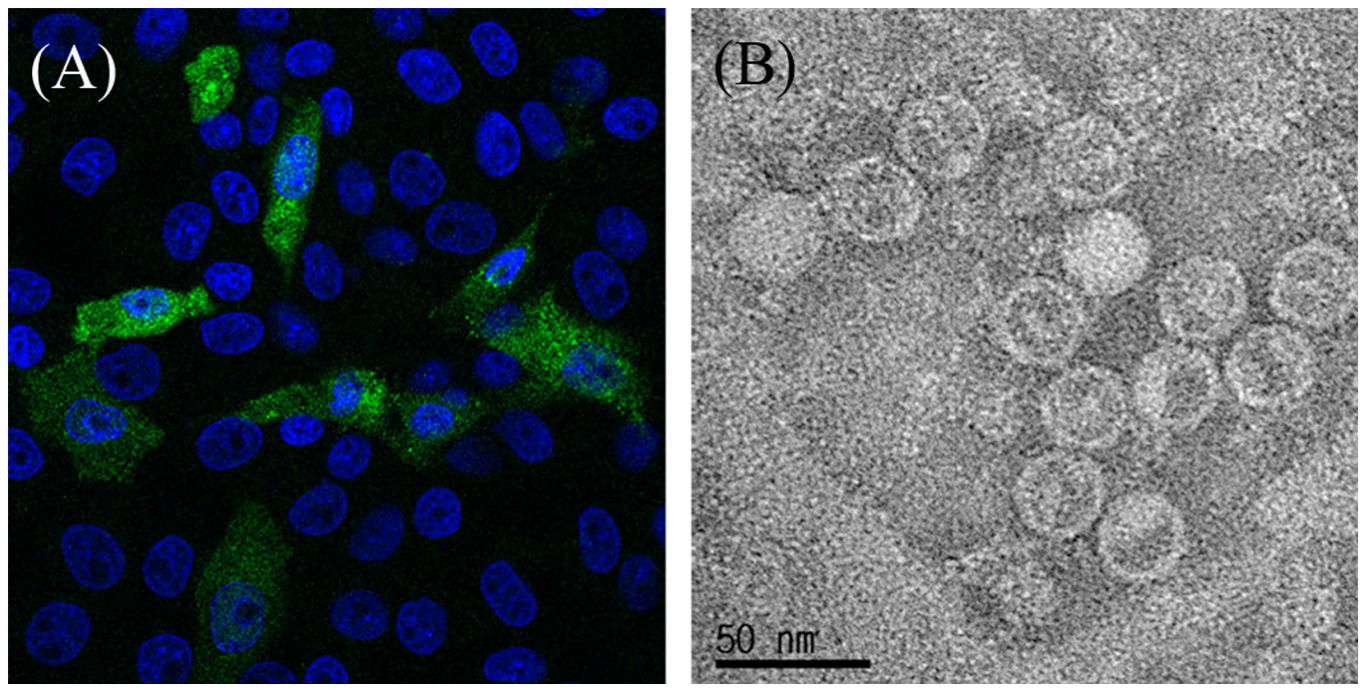
Identification and morphology of the porcine sapelovirus (PSV). (A) Immunofluorescence analysis of the PSV infected LLC-PK cells by laser confocal microscopy. The LLC-PK1 cells were incubated with mouse anti-153/5B5 monoclonal antibody, followed by staining with fluorescein-conjugated goat anti-mouse IgG antibody (green fluorescence). The nuclei were visualized by staining with DAPI (blue fluorescence). (B) Electron micrograph (EM) of cultured PSV strain KS05151. Virus pelleted by ultracentrifugation was stained with 1% phosphotungstic acid and sprayed onto coated EM grids.

Three Korean PSV strains were isolated from separate diarrhea fecal samples originating from three different farms and plaque purified. LLC-PK cells infected with each strain after 8 passages in LLC-PK cells showed CPE at day 1 post-inoculation characterized by shrinkage, rounding and detachment of cells (Figure 1A, 1B), and displayed PSV-specific cytoplasmic fluorescence in the indirect IFA using a monoclonal antibody against PSV capsid protein (Figure 1A). RT-PCR assays with a primer pair specific for the partial PSV VP1 coding region amplified a 636 bp amplicon from LLC-PC cells infected with each strain (Figure S1C). By transmission electron microscopy, negatively stained purified virus particles of each strain appeared spherical with a diameter of approximately 30 nm (Figure 1B). No other virus-like particles were observed. These results identified the isolated viruses as PSVs.

### Genome organization

The complete nucleotide sequences of the whole genome of the three Korean PSV strains were obtained and compared to the previously determined PSV sequences. In picornavirus RNAs, the two 5′ terminal UU residues are derived from the uridylylation of VPg to make VPg-pU-pU [28]. In the previously described PSV sequences, however, the two 5′ terminal residues were AC for the Chinese csh and English V13 strains, and UA for the Chinese YC2011 strain, suggestive of incomplete sequences. To obtain the correct 5′ terminal start residues, cDNA synthesis and then 5′ RACE PCR with 5′ RACE primer (Table S1 in File S1) were performed. Using this approach, the 5′ terminal residues were UU and the 5′ UTR length of three Korean strains was 25 nucleotides longer than that of Chinese YC2011 strain (Table S3 in File S1) [29]. In order to confirm this result, 5′RACE PCR was performed with another 5′RACE PCR primer (Table S1 in File S1) and the same 5′ terminal nucleotide residues, UU, were also observed.

**Figure 2:**
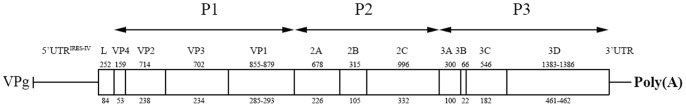
Genome organization of the porcine sapelovirus (PSV). The open reading frames are flanked on either side by UTRs. The numbers above or under each rectangle are the length of nucleotides or deduced amino acids. The length of VP1 and 3D regions are different among PSVs.

The length of the complete genomes of Korean PSV strains, excluding the poly(A) tail, was from 7,542 (KS04105 and KS055217) to 7,566 nucleotides (KS05151) (Table S3 in File S1). These sequences contained a single large ORF whose lengths were 6,966 nucleotides (strain V13; 2,322 amino acid polyprotein precursor) to 6,993 nucleotides (strains csh, KS05151, YC2011; 2,331 amino acid polyprotein precursor) (Table S3 in File S1). The predicted protease cleavage sites of the polyproteins, as determined from alignments with other picornaviruses are shown in Table 1. The polyprotein was predicted to be cleaved and processed into twelve mature peptides: L-VP4-VP2-VP3-VP1-2A-2B-2C-3A-3B-3C^Pro^-3D^Pol^ (Figure 2). The ORF sequence in the three Korean PSV strains was flanked by 5′ UTR which was 491 nucleotides long and by a 3′ UTR which was 82 nucleotides long (Table S3 in File S1).

**Table 1:** Location of putative cleavage sites in the porcine sapelovirus polyprotein.

Cleavage site	Amino acid sequence	Position of amino acid		
		1^a^	2^b^	3^c^
L/VP4	GNKPQ/GAYNH	84/85	84/85	84/85
VP4/VP2	GPSLK/APDKE	137/138	137/138	137/138
VP2/VP3	RQ/GFPVR	375/376	375/376	375/376
VP3/VP1	YQ/GD	609/610	609/610	609/610
VP1/2A	AEQL^a^^,^ b (ATQT^c^)/GPYE	902/903	894/895	894/895
2A/2B	HDWVQ/GLGQV	1128/1129	1120/1121	1120/1121
2B/2C	EPHKQ/GPSDW	1233/1234	1225/1226	1225/1226
2C/3A	DAIFQ/GPVQ	1565/1566	1557/1558	1557/1558
3A/3B	KQ/GAY	1665/1666	1657/1658	1657/1658
3B/3C	KAVVQ/GPDME	1687/1688	1679/1680	1679/1680
3C/3D	FVNKQ/GLITE	1869/1870	1861/1862	1861/1862
3D/	F/	2331/	2323/	2322/

Letters in bold represent conserved amino acid residues.

a YC2011, KS05151 and csh strains.

b KS055217 and KS04105 strains.

c V13 strain.

### Molecular and phylogenetic analyses

The complete genome sequence, excluding the poly(A) tail, and the polyprotein sequences of three Korean strains were compared with those of other known PSVs and representative picornavirus strains available in the GenBank database. The Korean PSV strains showed high nucleotide (84.7%–94.0%) and deduced amino acid (92.9%–98.3%) identities with the other PSV strains (Table 2), but showed relatively low nucleotide and polyprotein sequence identities with the avian and simian sapelovirus strains (Table 2).

**Table 2:** Comparison of complete nucleotide and deduced amino acid sequences between the Korean porcine sapelovirus (PSV) strains and other known picornavirus strains.

Strain^1^	Genus	Species	% nucleotide and deduced amino acid identities with strain:					
			KS04105		KS05151		KS055217	
			nt^2^	aa^3^	nt	aa	nt	aa
**KS04105**	***Sapelovirus***	**PSV**			**94.0**	**98.3**	**90.6**	**97.0**
**KS05151**	***Sapelovirus***	**PSV**	**94.0**	**98.3**			**89.2**	**96.5**
**KS055217**	***Sapelovirus***	**PSV**	**90.6**	**97.0**	**89.2**	**96.5**		
**V13**	***Sapelovirus***	**PSV**	**84.7**	**93.6**	**85.0**	**93.5**	**85.1**	**92.9**
**csh**	***Sapelovirus***	**PSV**	**88.0**	**97.1**	**88.8**	**97.4**	**87.5**	**95.6**
**YC2011**	***Sapelovirus***	**PSV**	**88.3**	**97.3**	**89.2**	**97.6**	**87.8**	**96.1**
TW90A	*Sapelovirus*	ASV^4^	44.2	43.3	44.1	43.4	43.8	43.8
2383	*Sapelovirus*	SSV^5^	56.3	55.0	56.2	54.8	56.7	54.9
F65	*Teschovirus*	PTV-1^6^	39.0	24.3	39.4	24.5	39.2	24.5
UKG/410/73	*Enterovirus*	PEV-9^7^	48.8	38.4	48.6	38.7	48.9	39.0
Ruckert	*Cardiovirus*	EMCV^8^	37.0	24.8	36.9	24.5	36.8	24.8
OTai	*Aphthovirus*	FMDV^9^	32.3	24.3	32.1	24.3	32.4	24.1
P1436/71	*Erbovirus*	ERBV-1^10^	36.5	23.3	36.5	23.1	36.4	23.5
Mahoney	*Enterovirus*	PV-1^11^	48.7	38.2	48.9	38.0	48.8	37.9
HM-175	*Hepatovirus*	HAV^12^	35.7	18.6	35.8	18.6	36.1	18.6
A846/88	*Kobuvirus*	AiV^13^	32.7	19.3	32.7	19.4	32.6	19.4
Gregory	*Parechovirus*	HPeV-1^14^	35.5	16.1	35.1	16.2	35.3	16.2
89	*Rhinovirus*	Human rhinovirus A	47.1	38.0	47.0	38.3	47.3	38.1
R85952	*Avihepatovirus*	DHAV^15^	36.5	16.7	36.4	16.3	36.3	16.6
SVV-001	*Senecavirus*	SVV^16^	36.4	23.7	36.6	23.6	36.6	23.8
Calnek	*Tremovirus*	AEV^17^	37.3	19.1	37.5	19.2	37.3	19.1

1 GenBank accession numbers of strains used are in Table S2.

2 The full-length nucleotide sequence identities among PSVs and other picornaviruses.

3 The full-length deduced amino acid sequence identities among PSVs and other picornaviruses.

4 ASV: avian sapelovirus.

5 SSV: simian sapelovirus.

6 PTV-1: porcine teschovirus serotype 1.

7 PEV-9: porcine enterovirus serotype 9.

8 EMCV: encephalomyocarditis virus.

9 FMDV: foot-and-mouth disease virus type O.

10 ERBV-1: Equine rhinitis B virus serotype 1.

11 PV-1: Poliovirus serotype 1(Human enterovirus C serotype).

12 HAV: Hepatitis A virus.

13 AiV: Aichi virus.

14 HPeV-1: Human parechovirus serotype 1.

15 DHVA: Duck hepatitis A virus.

16 SVV: Seneca Valley virus.

17 AEV: Avian encephalomyelitis virus.

**Figure 3:**
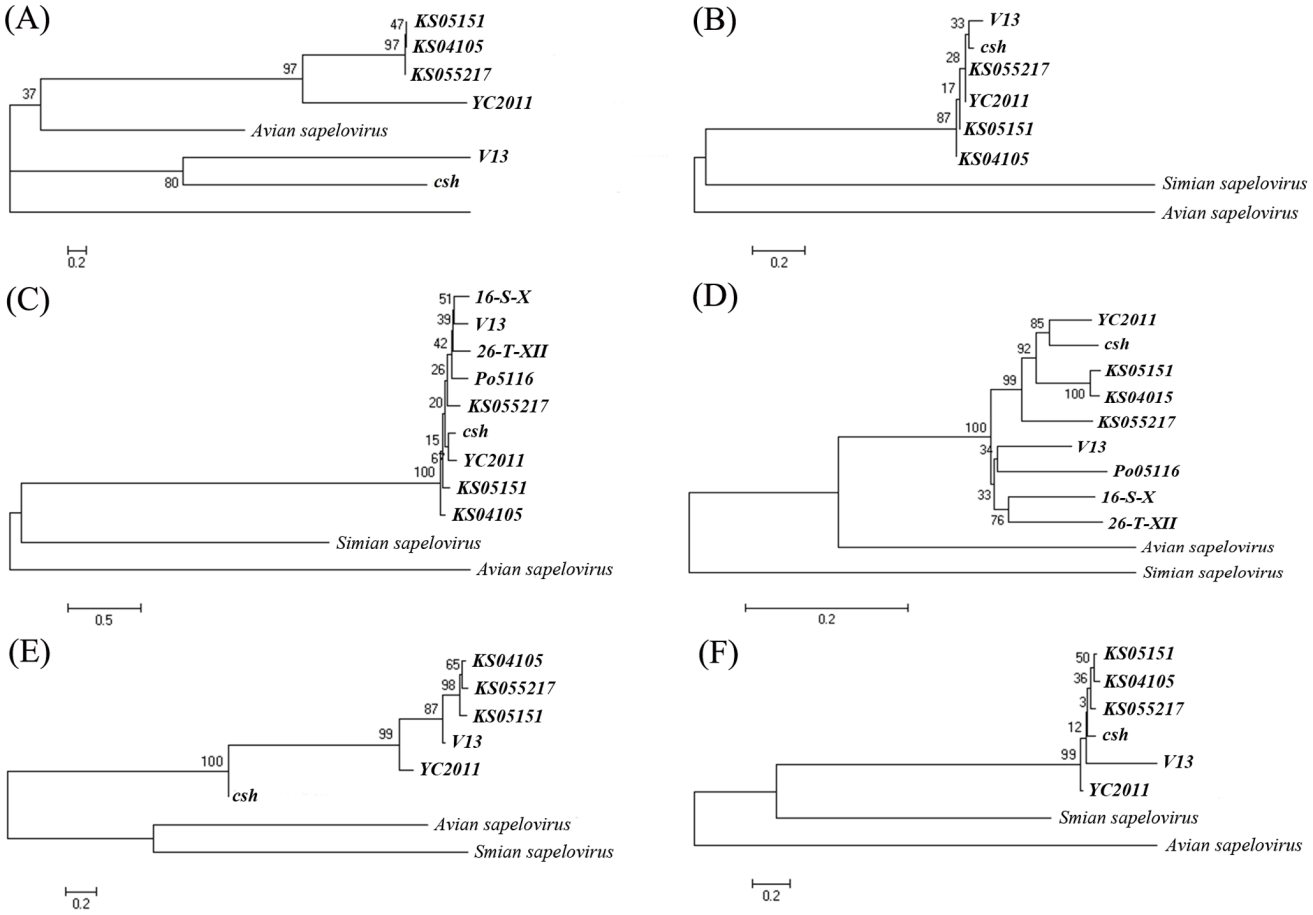
Sequence comparisons and phylogenetic analysis of Korea porcine sapelovirus strains. The phylogenetic tree of 5′ untranslated region (UTR) sequence (A), 3′UTR sequence (B), P1 nucleotide sequence (C), VP1 nucleotide sequence (D), P2 nucleotide sequence (E), and P3 nucleotide sequence (F) were constructed using the neighbor-joining method with 1,000 bootstrap replicates, and the branch length is indicated at each branch node.

Each of the major functional units in the genome, including the 5′ and 3′ UTRs, the capsid coding region (P1) and the regions encoding the non-structural proteins (P2 and P3) of PSVs, were phylogenetically analyzed (Figure 3). Representative simian and avian sapelovirus reference strains were included in each of the trees. The 5′ UTR sequences of three Korean PSV strains were in the same cluster and were more closely related to the English strain V13 than to the Chinese strains csh and YC2011 (Figure 3A). The 3′ UTR sequences of five strains (KS04105, KS055217, KS05151, V13, and YC2011) were 82 nucleotides long while that of Chinese strain csh was 68 nucleotides in length, possibly due to incomplete sequencing (Table S3 in File S1). However, they were phylogenetically very close (Figure 3B).

The leader protein sequences of all PSV strains were 252 nucleotides (84 amino acids) in length (Table S3 in File S1) and show high deduced amino acid identities (95.2–100%) (Table S4 in File S1). The PSV leader polypeptide lacked the catalytic residue motifs necessary for proteolytic activity and did not contain either a zinc-finger motif [Cys2 His2-like fold group] in the leader amino terminal region or a tyrosine-phosphorylation motif [KR]-x(2,3)-[ED]-x(2,3)-Y].

The nucleotide and deduced amino acid sequences of the PSV capsid region varied in length from 2430 to 2454 nucleotides (encoding 810 to 818 amino acids, Table S3 in File S1). To investigate the genetic relationships between the PSV strains, pairwise sequence identities were calculated for the deduced complete capsid protein sequences of all 6 PSV strains and for the sequences of each of the mature capsid proteins, VP1-VP4 (Table S4 in File S1). All PSV strains showed 88.4% to 97.7% nucleotide identities in the complete capsid coding sequences (Table S4 in File S1). The phylogenetic relationships are shown in Figure 3C. The PSV strains had 85.6% to 98.2% nucleotide identities within the VP1 coding sequences (Table S4 in File S1). The phylogenetic analysis for the VP1 proteins among PSV strains is shown in Figure 3D. The VP1 sequences of the KS05151, csh, and YC2011 strains encoded an additional 8 amino acids in comparison with other strains (Table S3 in File S1).

The P2 region of all PSV strains was 1989 nucleotides (663 amino acids) in length and P3 was 2235–2238 nucleotides (745–746 amino acids) long (Table S3 in File S1). Pairwise deduced amino acid sequence identities of P2 and P3 regions were shown to be very high, ranging from 94.1% to 99.5% and 95.9% to 99.0%, respectively (Table S4 in File S1).

### Analysis of the RNA structures within the 5′UTR

**Figure 4:**
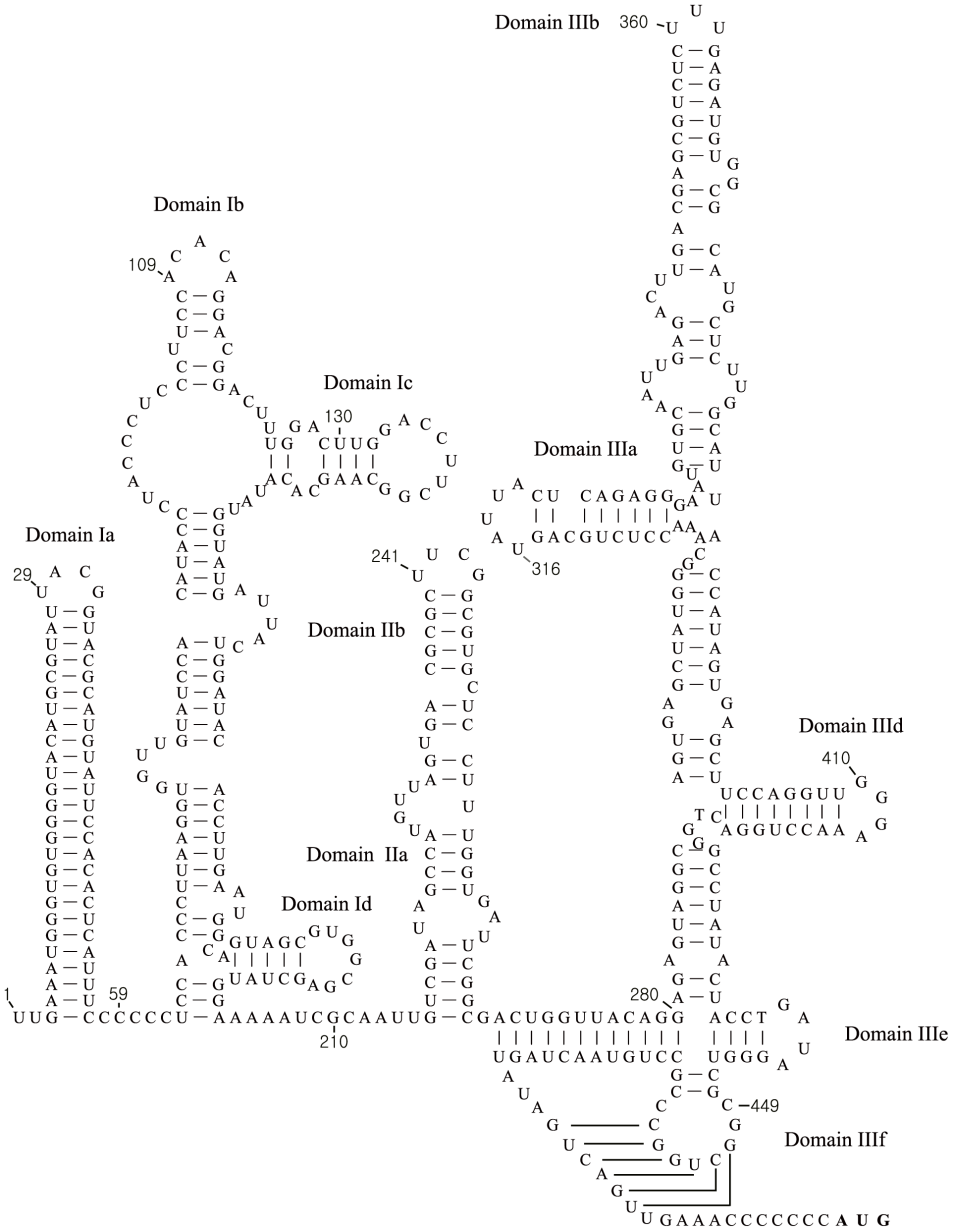
Sequences and structural features of 5′ untranslated region of the porcine sapelovirus KS05151 strain. At the extreme 5′ terminus are two stem-loop structures, labelled domains Ia and Ib. Secondary stem-loops include two smaller stem loop structures labelled Ic and Id. A secondary structure model for the domains II and III of the type IV internal ribosome entry site element is shown (this model is based on previously published studies [9], [29], [30]).

As described above, the use of 5′RACE allowed determination of the complete 5′UTR sequence of the Korean PSVs (some 491 nt in length); the 5′ UTR of these strains was 25 nucleotides longer than that of Chinese strain of PSV (YC2011). This allowed prediction of the secondary structure elements within the PSV 5′UTR (Figure 4). At the extreme 5′ terminus were two stem-loop structures, labelled domains Ia and Ib. The latter included two smaller stem loop structures, labelled Ic and Id. The 5′ UTR also contained two other domains, labelled domain II and domain III. These represent essential components of the IRES and are labelled in the same manner as other type IV IRES elements (related to that found in hepatitis C virus (HCV), the pestiviruses and certain picornaviruses, e.g. porcine teschovirus) [9], [30], [31]. Domain III contained multiple sub-domains including a pseudoknot (IIIf) and highly conserved stem-loops IIId and IIIe which, by analogy to the HCV IRES, interact directly with the 40S ribosomal subunit.

### Analysis of 3′UTR sequence

**Figure 5:**
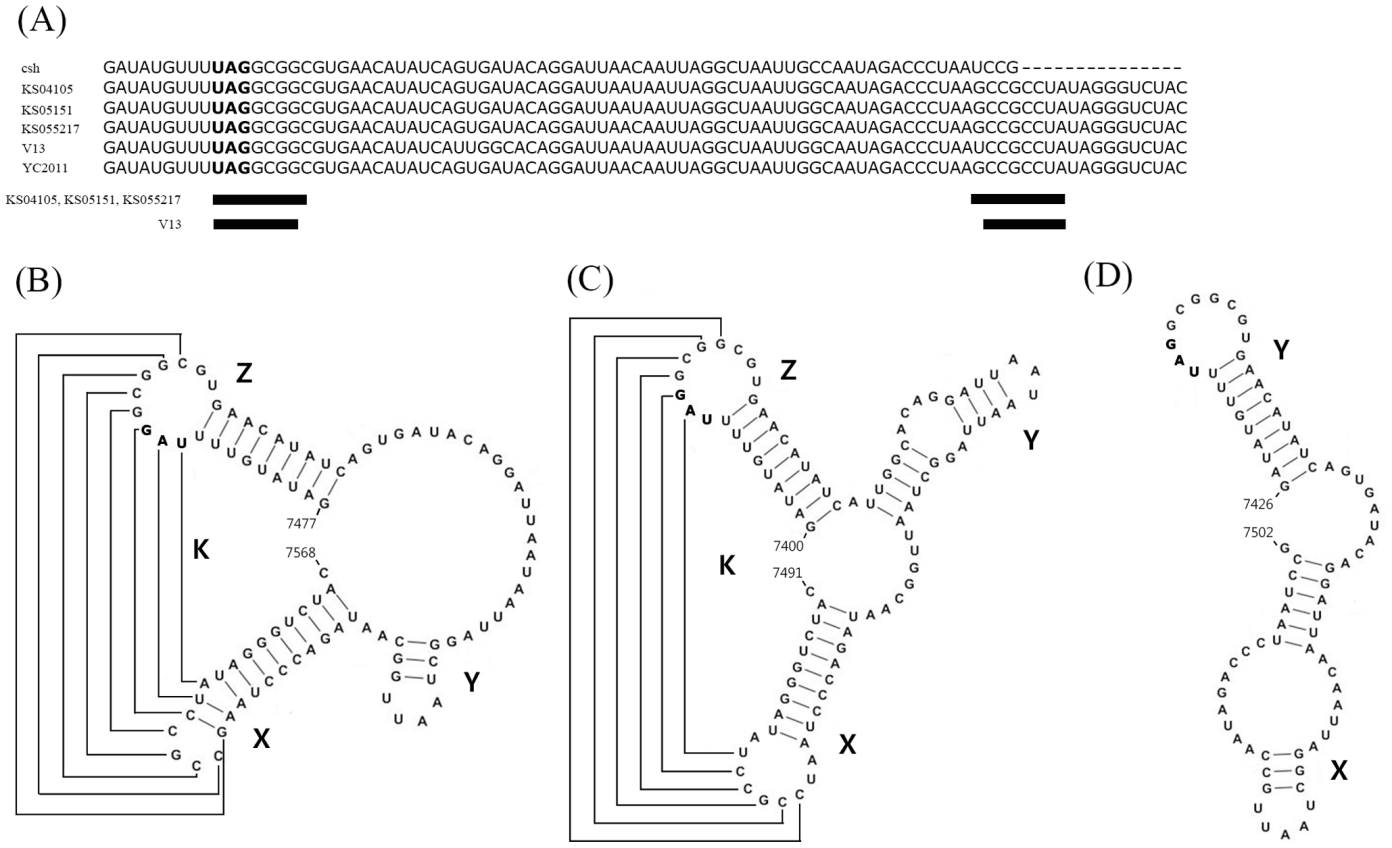
Sequences and structural features of 3′ untranslated region (UTR) of the porcine sapeloviruses. (A) The nucleotide sequences of the 3′ UTR were compared using the Clustal W methods. (B–D) Secondary and tertiary structures of 3′ UTR of strains KS04105, KS05151, KS055217 and YC2011 (B), strain V13 (C), and strain csh (D) were predicted by the CLC program. Proposed tertiary interactions between the loops of X and Z domains are shown by lines.

The PSV 3′UTR was highly conserved. Based on the size of the 3′UTR, the PSVs can be divided into a V13-like group (KS04105, KS05151, KS055217, YC2011 and V13 strains) which was 82 nucleotides long and the csh strain that was 62 nucleotides long (Figure 5A). Both of these sapelovirus subgroups possess two common domains that can form a stem-loop structure (Domains X and Y [Figure 5B–5D]). The V13-like group contained a third stem loop region (termed domain Z [Figure 5B, 5C]), located upstream of domain Y (Figure 5B, 5C). In the 3 Korean strains and YC2011 strain, an interaction of 8 nucleotides between the same sequence region within the loop of domain Z (nt 7485 to 7492 in the KS05151 strain) and the loop of domain X (nt 7549 to 7556 in the KS05151 strain), forming a loop-loop intramolecular “kissing” RNA interaction, appeared to be possible (Figure 5B). The V13 strain had a potential interaction of 7 nucleotides between the loops of domain Z (nt 7411 to 7416) and domain X (nt 7475 to 7480), forming a similar intramolecular “kissing” RNA interaction (Figure 5C). The csh-like group sequence was not predicted to form a loop-loop intramolecular “kissing” RNA interaction due to the lack of the domain Z (Figure 5A, 5D).

### Identification of a *cis*-acting RNA element (*CRE*)

**Figure 6:**
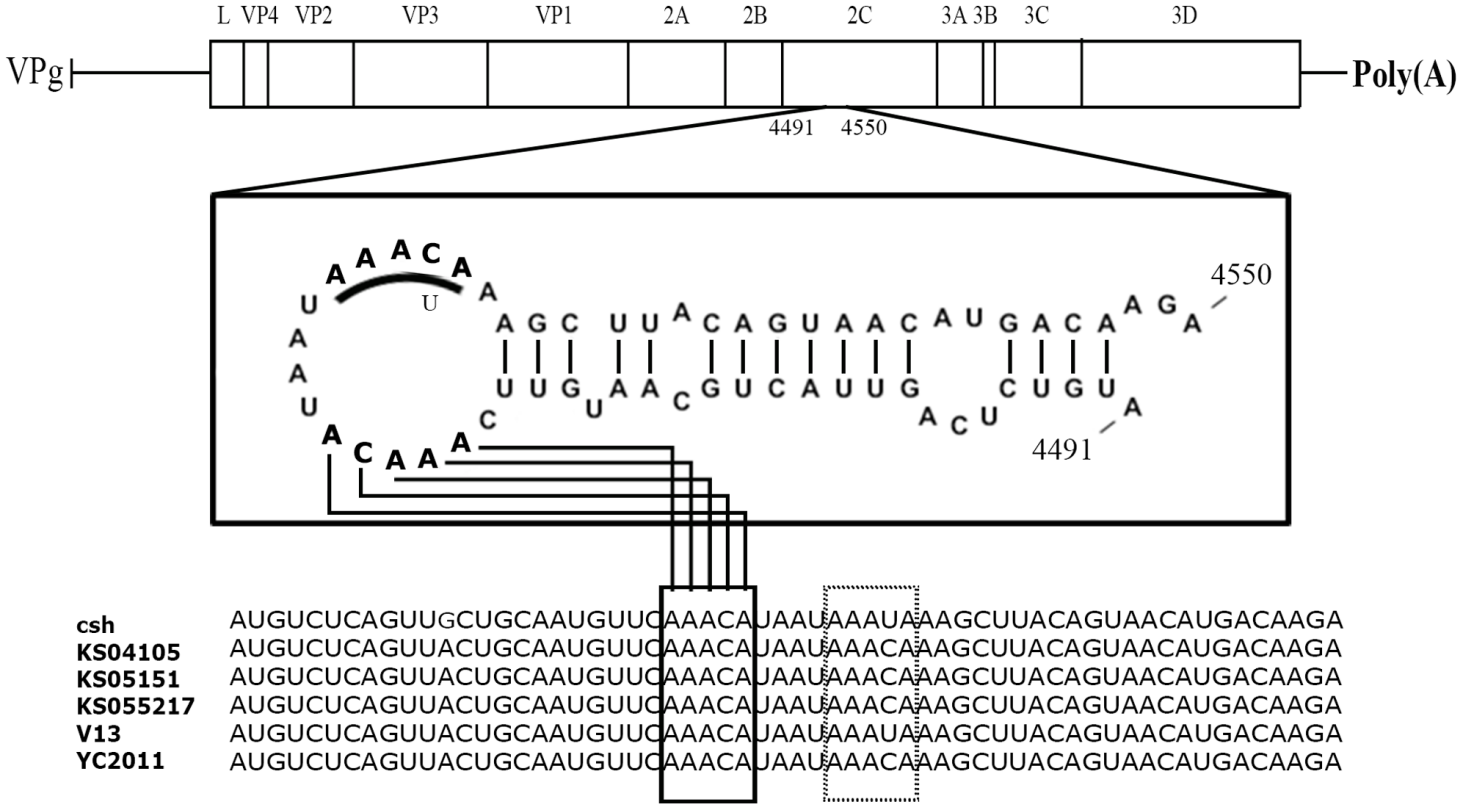
Sequences and structural feature of porcine sapelovirus *cis*-replication element (*CRE*). The first and second AAACA motifs are written in bold letter in the loop of the *CRE*.

The *CRE* is an essential element in picornavirus RNA replication [7], [32]–[35]. These relatively short elements can be located in different places within the genome; they act as the template for the uridylylation of VPg to form VPg-pU-pU and contain an essential motif AAAYA [36]. Analyses of the three Korean sequences (KS04105, KS05151, KS055217) and other known PSV strains including the Chinese (csh and YC2011) and English (V13) strains revealed the presence of several AAACA motifs in the genomes of these strains. As shown in Figure 6, however, a stem-loop structure with an exposed loop containing the AAACA motif was only found in the 2C coding sequence (*i.e.*, nucleotide 4491 to 4550 in the genome of KS05151). This hairpin loop included 16 nucleotides. Interestingly, the KS04015, KS05105, KS055217 and YC2011 strains had two AAACA motifs within this loop structure (Figure 6).

## Discussion

We report here the isolation of three Korean PSV strains, KS05151, KS04105 and KS055217 from porcine diarrhea specimens. The Korean PSV strains were identified as PSV by RT-PCR, IFA and TEM assays. The genome sequences of the Korean PSV strains were determined and proved to be the first complete genome sequences for PSVs. They have a genome organization typical for members of the genera *Cardiovirus*, *Aphthovirus*, *Erbovirus*, *Kobuvirus*, *Teschovirus* and *Senecavirus* [11]. Moreover, these strains had distinct 2A proteins from those of the *Enterovirus* genus and a 5′ UTR with a type IV IRES [12], [14], [15], [37].

The 5′ UTRs of picornaviruses are highly structured and contain an IRES that directs RNA translation by internal ribosome binding [8], [9]. Picornavirus IRES are currently divided into five distinct types by the primary sequence, secondary structure, location of the initiation codon and activity in different cell types [9], [10]. In a previous study [16], the IRES elements of PSV V13 strain and simian sapelovirus SV2 strain were found to be related functionally and structurally to the type IV IRES element from porcine teschovirus 1 and hepatitis C virus. Comparative sequence analysis of the Korean PSV strains with PSV V13 strain showed that the structural features of the IRES elements were well conserved in all PSV species including the Korean PSV strains but they lacked a domain IIIc [16].

At the 5′ UTR terminus, enteroviruses and rhinoviruses contain a cloverleaf structure which is involved in RNA replication [38]. In order to identify whether PSV species have a cloverleaf RNA structure at the 5′UTR, the complete 5′UTR needs to be known. However, the sequences of the known PSV strains including the English V13, and the Chinese csh and YC2011 strains lacked the 5′ terminal UU residues, which were necessary for picornavirus RNA replication [39]. Using 5′ RACE, an additional 25 nucleotides including 5′ terminal UU residues were identified compared to the recently sequenced Chinese YC2011 strain [29]. Unlike enteroviruses and rhinoviruses [38], the Korean PSV strains had no cloverleaf RNA structure at the 5′UTR. However, two conserved stem-loop motifs were present within the 5′-terminal 80 nucleotides (Figure 4). The role of these structures is not known but they may be expected to play a role in RNA replication analogous to the cloverleaf structure of the enteroviruses [38], [39]. Overall the 5′UTRs of PSVs were quite short, for example the poliovirus 5′UTR is about 740 nucleotides in length while the foot-and-mouth disease virus (FMDV) 5′UTR is over 1300 nucleotides [40].

The picornaviruses that have a L protein preceding the capsid region are members of the genera *Cardiovirus*, *Aphthovirus*, *Erbovirus*, *Kobuvirus*, *Teschovirus* and *Sapelovirus* [41]. In aphthoviruses and erboviruses, the L proteins are papain-like cysteine proteinases that are able to cleave at their own carboxy-terminus and also to induce the cleavage of the eukaryotic initiation factor (eIF) 4G, leading to the shut-off of host-cell protein synthesis [42], . The L protein of encephalomyocarditis virus (a cardiovirus) binds zinc, is phosphorylated during viral infection, and has been reported to affect the efficiency of genome translation [41]. The properties of the sapelovirus L protein are not known; it has neither the catalytic dyad (Cys and His), conserved in a papain-like thiol protease found in FMDV L protein [44], nor a putative zinc-binding motif, Cys-His-Cys-Cys, found in encephalomyocarditis virus [45]. Further studies are required to address the function of PSV L protein.

In general, picornavirus 3′ UTRs vary in length between 40 and 165 nucleotides. The length of the 3′ UTR in PSV is 82 nucleotides (strain V13, YC2011, KS04105, KS05151 and KS055217) although a shorter sequence (68 nucleotides) was described for the csh strain [20]. In a previous report [12], PSV strain V13 was predicted to include three stem-loop structures in the region of the 3′UTR using nine nucleotides of the terminal part of the 3D coding region. In the present study, the Korean viruses KS04105, KS05151 and KS055217 plus the Chinese YC2011 strain each appear to have these 3 stem-loop structures (X, Y and Z), but the csh strain showed only two stem-loop structures (X and Y) since the sequence was shorter. The domain Z, in which the stop codon (UGA) is located, is the most conserved region of the 3′UTR within PSVs, whereas domains Y and X were considered more variable regions (Figure 5B, 5C), as they show heterogeneity in both length and nucleotide sequence. The differences between the 5 different PSV strains and the csh strain appear attributable to an incomplete 3′ terminal sequence for the csh 3′ UTR region. Moreover, five strains, except for the strain csh, were predicted to have an intramolecular kissing RNA interaction between the X and Z domains. Due to the lack of 3′ terminal sequence of csh 3′ UTR region, no intramolecular kissing RNA interaction could be predicted (Fig. 5D). Interestingly, the V13 strain showed intramolecular interaction of 7 nucleotides, but the three Korean and one Chinese strains had intramolecular interaction of 8 nucleotides. The 3′ UTR plays an important role in picornavirus replication. Serial passage of mutant viruses in which such interactions were disrupted resulted in production of revertants in which the tertiary kissing interaction was restored, indicating the functional importance of the interaction in the enterovirus 3′UTR [46]–[50]. Further study is required to determine whether these regions are important for PSV replication using modifications of the relevant PSV nucleotide sequences.

A *CRE* of picornaviruses has been identified in six genera of *Picornaviridae* family; *Enterovirus* [6], [51], *Rhinovirus* [5], *Cardiovirus* [52], *Aphthovirus* [7], *Parechovirus* [4], and *Hepatovirus* [53]. However, no putative *CRE* has yet been reported for viruses in the *Sapelovirus*, *Kobuvirus*, *Erbovirus*, *Teschovirus*, *Avihepatovirus*, *Senecavirus* and *Tremovirus* genera. The location of the *CRE* in the genomic RNA varies between the picornavirus genera. It is located in the coding region for 2C in enteroviruses [6], [36], for VP1 in species B rhinoviruses [32], for VP2 in species C rhinoviruses [38] and cardioviruses [52], for VP0 in parechoviruses [4] and for 3D of hepatoviruses [54]. In FMDV, the *CRE* is located just upstream of the IRES [7], [35]. In the present study, a putative *CRE* was located in the 2C coding region of all PSV strains. Generally, the AAACA motif is located in the loop of picornavirus *CREs* and the first and second A residues are involved in providing a template for the addition of uridine onto VPg [38], [39], [53], [55], [56]. In contrast to other known *CREs*, the PSV strains had two copies of the AAACA motif within the sequence C**AAACA**TAAT**AAACA**A. This indicated that one or both of these AAACA motifs may be involved in being a template for the addition of uridine onto VPg. Future functional analyses are needed to identify whether one or both motifs are templates for the uridylylation of VPg to make VPg-pU-pU_OH_.

In this study, we characterized the structural features of three Korean PSV strains in comparison with the other known PSV strains. All PSV strains showed the typical picornavirus genome organization. We have identified putative RNA structures in the 5′UTR and 3′ UTR plus a *CRE* in the 2C coding sequence. Interestingly, the structural features of the *CRE* in the 2C coding sequence and of the 3′UTR were different between the strains circulating in the recent and past decades. These first complete genome data for PSV (for the Korean PSV strains KS05151, KS04105 and KS055217) will facilitate future investigations concerning the molecular pathogenesis and evolutionary characteristics of this virus.

## Supporting Information

Figure S1 **Phase contrast photomicrographs of control and infected LLC-PK1 cells, and RT-PCR assay for detecting porcine sapelovirus (PSV) VP1 coding region.** (A) Mock-inoculated control cells. (B) Cells at 1 day after infection with Korean PSV strain KS05151. Note the shrinking and rounding up of the infected cells. Microscope settings Ocular: 10; Lens: 10X. Scale bar, 200 µm. (C) RT-PCR with primers specific for part of the PSV VP1 coding region generated the expected 636 bp amplicons. M: size marker. N: mock-infected LLC-PK cells. Lanes 1–3: KS04105, KS05151, and KS055217 strains.
(TIF)

File S1 **Supplementary Tables. Table S1.** Oligonucleotide primers for amplifying and sequencing of porcine sapelovirus strains. **Table S2.** Strains of picornaviruses and their GenBank accession numbers used in this study. **Table S3.** The length of 5′ untranslated region, each part of the open reading frame, 3′ untranslated region and the complete genome excepting the poly(A) tail. **Table S4.** Comparison of nucleotide/deduced amino acid sequences between the porcine sapelovirus strains.
(DOC)

## References

[1] Le Gall O, Christian P, Fauquet CM, King AM, Knowles NJ, et al (2008) Picornavirales, a proposed order of positive-sense single-stranded RNA viruses with a pseudo-T = 3 virion architecture. Arch Virol 153: 715–727. 18293057 10.1007/s00705-008-0041-x

[2] Racaniello VR (2007) Picornaviridae: the viruses and their replication. In: Knipe DM, Howley PM, editors. Fields Virology. Philadelphia: Lippincott Williams & Wilkins. pp. 795–838.

[3] Steil BP, Barton DJ (2009) Cis-active RNA elements (CREs) and picornavirus RNA replication. Virus Res 139: 240–252. 18773930 10.1016/j.virusres.2008.07.027 PMC2692539

[4] Al-Sunaidi M, Williams CH, Hughes PJ, Schnurr DP, Stanway G (2007) Analysis of a new human parechovirus allows the definition of parechovirus types and the identification of RNA structural domains. J Virol 81: 1013–1021. 17005640 10.1128/JVI.00584-06 PMC1797470

[5] Gerber K, Wimmer E, Paul AV (2001) Biochemical and genetic studies of the initiation of human rhinovirus 2 RNA replication: identification of a cis-replicating element in the coding sequence of 2A(pro). J Virol 75: 10979–10990. 11602738 10.1128/JVI.75.22.10979-10990.2001 PMC114678

[6] Goodfellow I, Chaudhry Y, Richardson A, Meredith J, Almond JW, et al (2000) Identification of a cis-acting replication element within the poliovirus coding region. J Virol 74: 4590–4600. 10775595 10.1128/jvi.74.10.4590-4600.2000 PMC111979

[7] Mason PW, Bezborodova SV, Henry TM (2002) Identification and characterization of a cis-acting replication element (cre) adjacent to the internal ribosome entry site of foot-and-mouth disease virus. J Virol 76: 9686–9694. 12208947 10.1128/JVI.76.19.9686-9694.2002 PMC136496

[8] Belsham GJ, Jackson RJ (2000) Translation initiation on picornavirus RNA. In: Sonenberg N, Hershey JWB, Mathews MB, editors. Translational Control of Gene Expression. NY: Cold Spring Harbor Laboratory Press. pp. 869–900.

[9] Belsham GJ (2009) Divergent picornavirus IRES elements. Virus Res 139: 183–192. 18675861 10.1016/j.virusres.2008.07.001

[10] Sweeney TR, Dhote V, Yu Y, Hellen CU (2012) A distinct class of internal ribosomal entry site in members of the Kobuvirus and proposed Salivirus and Paraturdivirus genera of the Picornaviridae. J Virol 86: 1468–1486. 22114340 10.1128/JVI.05862-11 PMC3264332

[11] Hales LM, Knowles NJ, Reddy PS, Xu L, Hay C, et al (2008) Complete genome sequence analysis of Seneca Valley virus-001, a novel oncolytic picornavirus. J Gen Virol 89: 1265–1275. 18420805 10.1099/vir.0.83570-0

[12] Krumbholz A, Dauber M, Henke A, Birch-Hirschfeld E, Knowles NJ, et al (2002) Sequencing of porcine enterovirus group II and III reveals unique features of both virus groups. J Virol 76: 5813–5821. 11992011 10.1128/JVI.76.11.5813-5821.2002 PMC137026

[13] Oberste MS, Maher K, Pallansch MA (2003) Genomic evidence that simian virus 2 and six other simian picornaviruses represent a new genus in Picornaviridae. Virology 314: 283–293. 14517081 10.1016/s0042-6822(03)00420-3

[14] Tseng CH, Tsai HJ (2007) Sequence analysis of a duck picornavirus isolate indicated that it together with porcine enterovirus type 8 and simian picornavirus type 2 should be assigned to a new picornavirus genus. Virus Res 129: 104–114. 17686542 10.1016/j.virusres.2007.06.023

[15] Oberste MS, Maher K, Pallansch MA (2002) Molecular phylogeny and proposed classification of the simian picornaviruses. J Virol 76: 1244–1251. 11773400 10.1128/JVI.76.3.1244-1251.2002 PMC135860

[16] Chard LS, Bordeleau ME, Pelletier J, Tanaka J, Belsham GJ (2006) Hepatitis C virus-related internal ribosome entry sites are found in multiple genera of the family Picornaviridae. J Gen Virol 87: 927–936. 16528042 10.1099/vir.0.81546-0

[17] International Committee on Taxonomy of viruses, ICTV (2013) Master species list. Available: http://talk.ictvonline.org. Accessed 2014 May 20.

[18] Knowles NJ, Hovi T, Hyypiä T, King AMQ, Lindberg AM, et al.. (2012) Picornaviridae. In: King AMQ, Adams MJ, Carstens EB, Lefkowitz EJ, editors. Virus taxonomy: classification and nomenclature of viruses: Ninth Report of the International Committee on Taxonomy of Viruses. San Diego: Elsevier. pp. 855–880.

[19] Knowles NJ (2006) Porcine enteric picornaviruses. In: Straw BE, Zimmerman JJ, D’Allaire S, Taylor DJ, editors. Diseases of swine. Oxford: Blackwell. pp. 337–345.

[20] Lan D, Ji W, Yang S, Cui L, Yang Z, et al (2011) Isolation and characterization of the first Chinese porcine sapeloviruse strain. Arch Virol 156: 1567–1574. 21618029 10.1007/s00705-011-1035-7

[21] Abe M, Ito N, Sakai K, Kaku Y, Oba M, et al (2011) A novel sapelovirus-like virus isolation from wild boar. Virus Genes 43: 243–248. 21643767 10.1007/s11262-011-0628-2

[22] Buitrago D, Cano-Gómez C, Agüero M, Fernandez-Pacheco P, Gómez-Tejedor C, et al (2010) A survey of porcine picornaviruses and adenoviruses in fecal samples in Spain. J Vet Diagn Invest 22: 763–766. 20807938 10.1177/104063871002200519

[23] Honda E, Hattori I, Oohara Y, Taniquchi T, Ariyama K, et al (1990) Sero- and CPE-types of porcine enteroviruses isolated from healthy and diarrheal pigs: possible association of CPE type II with diarrhea. Nihon Juigaku Zasshi 52: 85–90. 2156103 10.1292/jvms1939.52.85

[24] Prodělalová J (2012) The survey of porcine teschoviruses, sapeloviruses and enteroviruses B infecting domestic pigs and wild boars in the Czech Republic between 2005 and 2011. Infect Genet Evol 12: 1447–1451. 22579481 10.1016/j.meegid.2012.04.025

[25] Sozzi E, Barbieri I, Lavazza A, Lelli D, Moreno A, et al (2010) Molecular characterization and phylogenetic analysis of VP1 of porcine enteric picornaviruses isolates in Italy. Transbound Emerg Dis 57: 434–442. 21040508 10.1111/j.1865-1682.2010.01170.x

[26] Son KY, Kim DS, Matthijnssens J, Kwon HJ, Park JG, et al (2014) Molecular epidemiology of Korean porcine sapeloviruses. Arch Virol 159: 1175–1180. 24232913 10.1007/s00705-013-1901-6 PMC7087272

[27] Tamura K, Peterson D, Peterson N, Stecher G, Nei M, et al (2011) MEGA5: molecular evolutionary genetics analysis using maximum likelihood, evolutionary distance, and maximum parsimony methods. Mol Biol Evol 28: 2731–2739. 21546353 10.1093/molbev/msr121 PMC3203626

[28] Crawford NM, Baltimore D (1983) Genome-linked protein VPg of poliovirus is present as free VPg and VPg-pUpU in poliovirus-infected cells. Proc Natl Acad Sci U S A 80: 7452–7455. 6324173 10.1073/pnas.80.24.7452 PMC389969

[29] Chen J, Chen F, Zhou Q, Li W, Song Y, et al (2012) Complete genome sequence of a novel porcine Sapelovirus strain YC2011 isolated from piglets with diarrhea. J Virol 86: 10898. 22966190 10.1128/JVI.01799-12 PMC3457332

[30] Pisarev AV, Chard LS, Kaku Y, Johns HL, Shatsky IN, et al (2004) Functional and structural similarities between the internal ribosome entry sites of hepatitis C virus and porcine teschovirus, a picornavirus. J Virol 78: 4487–4497. 15078929 10.1128/JVI.78.9.4487-4497.2004 PMC387690

[31] Hellen CU, de Breyne S (2007) A distinct group of hepacivirus/pestivirus-like internal ribosomal entry sites in members of diverse picornavirus genera: evidence for modular exchange of functional noncoding RNA elements by recombination. J Virol 81: 5850–5863. 17392358 10.1128/JVI.02403-06 PMC1900287

[32] McKnight KL, Lemon SM (1998) The rhinovirus type 14 genome contains an internally located RNA structure that is required for viral replication. RNA 4: 1569–1584. 9848654 10.1017/s1355838298981006 PMC1369726

[33] Paul AV (2002) Possible unifying mechanism of picornavirus genome replication. In: Semler BL, Wimmer E, editors. Molecular Biology of Picornaviruses. Washington, DC: ASM Press. pp. 227–246.

[34] Goodfellow IG, Polacek C, Andino R, Evans DJ (2003) The poliovirus 2C cis-acting replication element-mediated uridylylation of VPg is not required for synthesis of negative-sense genomes. J Gen Virol 84: 2359–2363. 12917456 10.1099/vir.0.19132-0

[35] Nayak A, Goodfellow IG, Belsham GJ (2005) Factors required for the uridylylation of the foot-and-mouth disease virus 3B1, 3B2, and 3B3 peptides by the RNA-dependent RNA polymerase (3Dpol) in vitro. J Virol 79: 7698–7706. 15919922 10.1128/JVI.79.12.7698-7706.2005 PMC1143669

[36] Paul AV, Yin J, Mugavero J, Rieder E, Liu Y, et al (2003) A “slide-back” mechanism for the initiation of protein-primed RNA synthesis by the RNA polymerase of poliovirus. J Biol Chem 278: 43951–43960. 12937178 10.1074/jbc.M307441200

[37] Chard LS, Kaku Y, Jones B, Nayak A, Belsham GJ (2006) Functional analyses of RNA structures shared between the internal ribosome entry sites of hepatitis C virus and the picornavirus porcine teschovirus 1 Talfan. J Virol 80: 1271–1279. 16415004 10.1128/JVI.80.3.1271-1279.2006 PMC1346926

[38] Cordey S, Gerlach D, Junier T, Zdobnov EM, Kaiser L, et al (2008) The cis-acting replication elements define human enterovirus and rhinovirus species. RNA 14: 1568–1578. 18541697 10.1261/rna.1031408 PMC2491478

[39] Steil BP, Barton DJ (2009) Cis-active RNA elements (CREs) and picornavirus RNA replication. Virus Res 139: 240–252. 18773930 10.1016/j.virusres.2008.07.027 PMC2692539

[40] Forss S, Strebel K, Beck E, Schaller H (1984) Nucleotide sequence and genome organization of foot-and-mouth disease virus. Nucleic Acids Res 12: 6587–6601. 6089122 10.1093/nar/12.16.6587 PMC320098

[41] Dvorak CM, Hall DJ, Hill M, Riddle M, Pranter A, et al (2001) Leader protein of encephalomyocarditis virus binds zinc, is phosphorylated during viral infection, and affects the efficiency of genome translation. Virology 290: 261–271. 11883190 10.1006/viro.2001.1193

[42] Devaney MA, Vakharia VN, Lloyd RE, Ehrenfeld E, Grubman MJ (1988) Leader protein of foot-and-mouth disease virus is required for cleavage of the p220 component of the cap-binding protein complex. J Virol 62: 4407–4409. 2845152 10.1128/jvi.62.11.4407-4409.1988 PMC253884

[43] Gradi A, Foeger N, Strong R, Svitkin YV, Sonenberg N, et al (2004) Cleavage of eukaryotic translation initiation factor 4GII within foot-and-mouth disease virus-infected cells: identification of the L-protease cleavage site in vitro. J Virol 78: 3271–3278. 15016848 10.1128/JVI.78.7.3271-3278.2004 PMC371048

[44] Gorbalenya AE, Koonin EV, Lai MM (1991) Putative papain-related thiol proteases of positive-strand RNA viruses. Identification of rubi- and aphthovirus preoteases and delineation of a novel conserved domain associated with proteases of rubi-, alpha- and coronaviruses. FEBS Lett 19: 201–205. 10.1016/0014-5793(91)81034-6 PMC7130274 1652473

[45] Chen CY, Sarnow P (1995) Initiation of protein synthesis by the eukaryotic translational apparatus on circular RNAs. Science 268: 415–417. 7536344 10.1126/science.7536344

[46] Pilipenko EV, Poperechny KV, Maslova SV, Melchers WJ, Slot HJ, et al (1996) Cis-element, oriR, involved in the initiation of (-) strand poliovirus RNA: a quasi-globular multi-domain RNA structure maintained by tertiary (‘kissing’) interactions. EMBO J 15: 5428–5436. 8895586 PMC452285

[47] Melchers WJ, Hoenderop JG, Bruins Slot HJ, Pleij CW, Pilipenko EV, et al (1997) Kissing of the two predominant hairpin loops in the coxsackie B virus 3′ untranslated region is the essential structural feature of the origin of replication required for negative-strand RNA synthesis. J Virol 71: 686–696. 8985400 10.1128/jvi.71.1.686-696.1997 PMC191101

[48] Melchers WJ, Bakkers JM, Bruins Slot HJ, Galama JM, Agol VI, et al (2000) Cross-talk between orientation-dependent recognition determinants of a complex control RNA element, the enterovirus oriR. RNA 6: 976–987. 10917594 10.1017/s1355838200000480 PMC1369974

[49] Wang J, Bakkers JM, Galama JM, Bruins Slot HJ, Pilipenko EV, et al (1999) Structural requirements of the higher order RNA kissing element in the enteroviral 3′UTR. Nucleic Acids Res 27: 485–490. 9862969 10.1093/nar/27.2.485 PMC148204

[50] van Ooij MJ, Polacek C, Glaudemans DH, Kuijpers J, van Kuppeveld FJ, et al (2006) Polyadenylation of genomic RNA and initiation of antigenomic RNA in a positive-strand RNA virus are controlled by the same cis-element. Nucleic Acids Res 34: 2953–2965. 16738134 10.1093/nar/gkl349 PMC1474053

[51] Paul AV, Rieder E, Kim DW, van Boom JH, Wimmer E (2000) Identification of an RNA hairpin in poliovirus RNA that serves as the primary template in the in vitro uridylylation of VPg. J Virol 74: 10359–10370. 11044080 10.1128/jvi.74.22.10359-10370.2000 PMC110910

[52] Lobert PE, Escriou N, Ruelle J, Michiels T (1999) A coding RNA sequence acts as a replication signal in cardioviruses. Proc Natl Acad Sci U S A 96: 11560–11565. 10500216 10.1073/pnas.96.20.11560 PMC18073

[53] Yang Y, Rijnbrand R, McKnight KL, Wimmer E, Paul A, et al (2002) Sequence requirements for viral RNA replication and VPg uridylylation directed by the internal cis-acting replication element (cre) of human rhinovirus type 14. J Virol 76: 7485–7494. 12097561 10.1128/JVI.76.15.7485-7494.2002 PMC136355

[54] Yang Y, Rijnbrand R, Watowich S, Lemon SM (2004) Genetic evidence for an interaction between a picornaviral cis-acting RNA replication element and 3CD protein. J Biol Chem 279: 12659–12667. 14711816 10.1074/jbc.M312992200

[55] Steil BP, Barton DJ (2009) Cis-active RNA elements (CREs) and picornavirus RNA replication. Virus Res 139: 240–252. 18773930 10.1016/j.virusres.2008.07.027 PMC2692539

[56] Thiviyanathan V, Yang Y, Kaluarachchi K, Rijnbrand R, Gorenstein DG, et al (2004) High-resolution structure of a picornavirial internal cis-acting RNA replication element (cre). Proc Natl Acad Sci U S A 101: 12688–12693. 15314212 10.1073/pnas.0403079101 PMC515117

