# Supplementary figures and images for: Full-Length Genomic Analysis of Korean Porcine Sapelovirus Strains
Source: PLoS One. 2014 Sep 17;9(9):e107860. doi: 10.1371/journal.pone.0107860 (PMC4168140; doi:10.1371/journal.pone.0107860)

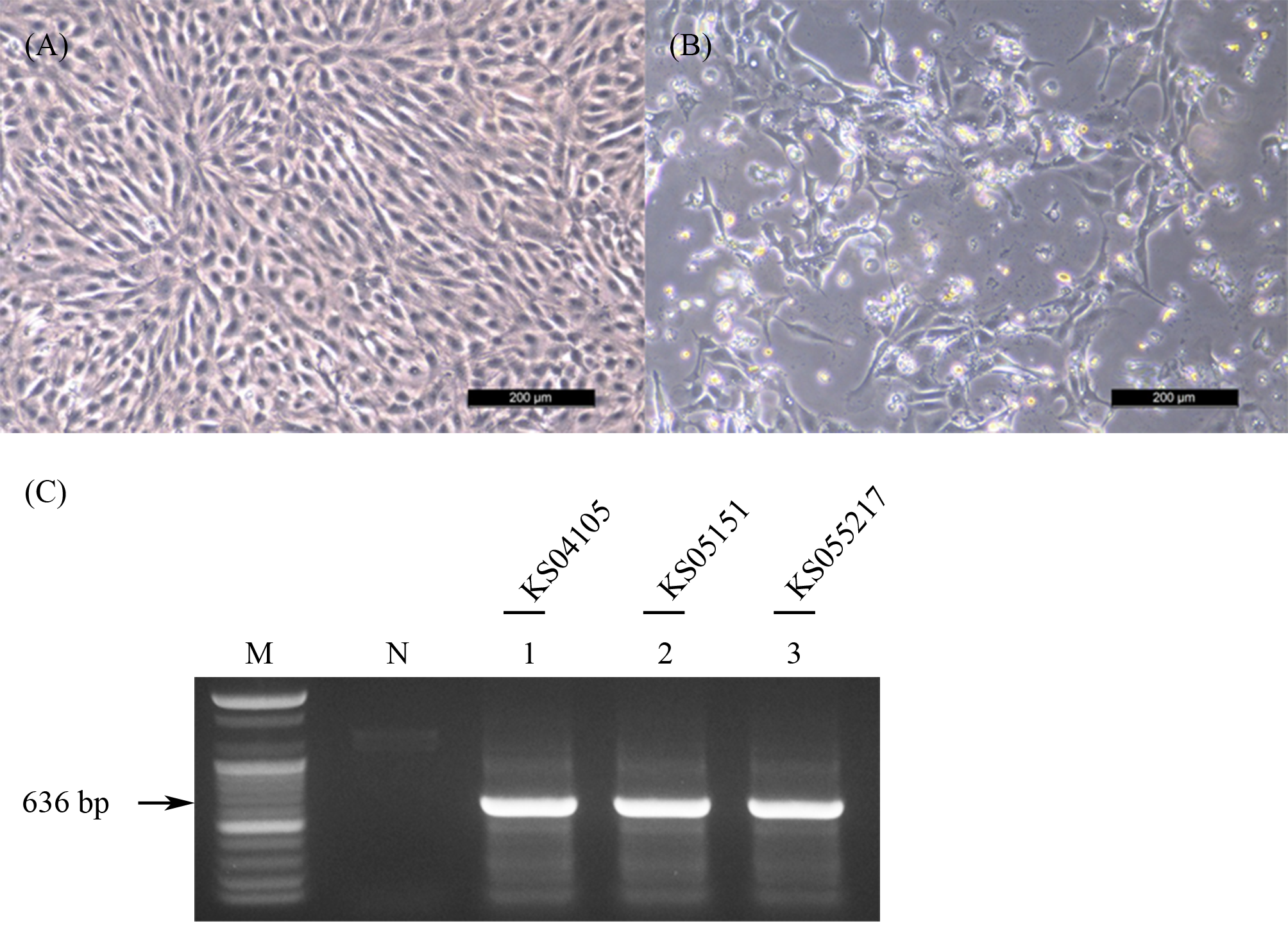

Supplement: Figure S1 — Phase contrast photomicrographs of control and infected LLC-PK1 cells, and RT-PCR assay for detecting porcine sapelovirus (PSV) VP1 coding region. (A) Mock-inoculated control cells. (B) Cells at 1 day after infection with Korean PSV strain KS05151. Note the shrinking and rounding up of the infected cells. Microscope settings Ocular: 10; Lens: 10X. Scale bar, 200 µm. (C) RT-PCR with primers specific for part of the PSV VP1 coding region generated the expected 636 bp amplicons. M: size marker. N: mock-infected LLC-PK cells. Lanes 1–3: KS04105, KS05151, and KS055217 strains. (TIF) [file pone.0107860.s001.tif]
