# Supplementary material for: Full-Length Genomic Analysis of Korean Porcine Sapelovirus Strains
Source: PLoS One. 2014 Sep 17;9(9):e107860. doi: 10.1371/journal.pone.0107860 (PMC4168140; doi:10.1371/journal.pone.0107860)
Supplement: File S1 — Supplementary Tables. Table S1. Oligonucleotide primers for amplifying and sequencing of porcine sapelovirus strains. Table S2. Strains of picornaviruses and their GenBank accession numbers used in this study. Table S3. The length of 5′ untranslated region, each part of the open reading frame, 3′ untranslated region and the complete genome excepting the poly(A) tail. Table S4. Comparison of nucleotide/deduced amino acid sequences between the porcine sapelovirus strains. (DOC) [file pone.0107860.s002.doc]

**Table S1.** Oligonucleotide primers for amplifying and sequencing of porcine sapelovirus strains.

| Primer name | Primer sequence (5’-3’) | Region (nt) | Size (bp) | References or sources |
| --- | --- | --- | --- | --- |
| 5’RACE^a^ | CAGGTTTCCCAACCTGGAAGCTCAC | 345-371 | 371 | In this study |
| 5’RACE^a^ | CCACACTCATTTCCCCCCTCCACCC | 21-45 | 45 | In this study |
| 3’RACE^b^ | CTATTGGCATGGCACAATGGTGAGG | 7281-7305 | 197 | In this study |
| VP1F | AGGATGTGGTGCAAGCAAGCAT | 2282-2303 | 636 | [26] |
| VP1R | AGGCAGCACCGTTCTGGTCAA | 2897-2917 |  | [26] |
| PSV1F | AGCGTGGCGAGCTATGGAAAAATCGCAATT | 139-168 | 833 | In this study |
| PSV1R | CCGCCTTGGCTGCCTCTTGTGTTGTTAT | 971-944 |  | In this study |
| PSV2F | AGGCACCTGATAAGGAGGAAGAAGG | 876-901 | 656 | In this study |
| PSV2R | GTTATGGGAACAAAGGGAGAGGCACC | 1531-1506 |  | In this study |
| PSV3F | GCACACAATTTTGTTACATTAGT | 1449-1471 | 722 | In this study |
| PSV3R | TGAAATATGGTCACCCATCCATTGTATGA | 2170-2142 |  | In this study |
| PSV4F | G GGATATAGGTTTACAATCAACATGC | 2050-2075 | 876 | In this study |
| PSV4R | GGTCAAAGTTACCATCACCATCATA | 2925-2901 |  | In this study |
| PSV5F | ATACCCTTTATGTCTGTTTGTAATTATTA | 2862-2890 | 809 | In this study |
| PSV5R | GCTCCACAGAAGCCATTATCAGCATC | 3670-3645 |  | In this study |
| PSV6F | TACTCAGGACCCAAACCATTTAGA | 3564-3587 | 946 | In this study |
| PSV6R | TAAGCTTTATTTATTATGTTTGAACATTGC | 4510-4481 |  | In this study |
| PSV7F | AATATGGTCACAAATTTAATCTACAA | 4441-4466 | 868 | In this study |
| PSV7R | CCAGCCTTTTCTAACTCATCTATTATAA | 5308-5281 |  | In this study |
| PSV8F | CATGATGTGATGGAACTGGTTAGGTGCAC | 5241-5269 | 729 | In this study |
| PSV8R | ACACCTCCACACTGTCCCATTTT | 5969-5952 |  | In this study |
| PSV9F | TGGATTTCTTAACCTATCCATGACCCC | 5891-5917 | 740 | In this study |
| PSV9R | TCATCATTATGGTATCATTGAGTGATGA | 6630-6603 |  | In this study |
| PSV10F | TGAAGATGGG CAAGACCAGG GTTATTGA | 6571-6598 | 841 | In this study |
| PSV10R | TACGCAGCTGAGAGTAAGGTGGTAATATGA | 7382-7411 |  | In this study |

^a^Primer for 5’ cDNA synthesis.

^b^Primer for 3’ cDNA synthesis.

**Table S2.** Strains of picornaviruses and their GenBank accession numbers used in this study.

| Genus | Species | Strain | Accession number |
| --- | --- | --- | --- |
| *Sapelovirus* | Porcine sapelovirus | KS04105 | KJ821019 |
| *Sapelovirus* | Porcine sapelovirus | KS055217 | KJ821020 |
| *Sapelovirus* | Porcine sapelovirus | KS05151 | KJ821021 |
| *Sapelovirus* | Porcine sapelovirus | V13 | NC_003987.1 |
| *Sapelovirus* | Porcine sapelovirus | csh | HQ875059.1 |
| *Sapelovirus* | Porcine sapelovirus | YC2011 | JX286666.1 |
| *Sapelovirus* | Porcine sapelovirus | 16-S-X | AY392543 |
| *Sapelovirus* | Porcine sapelovirus | 26-T-XII | AY392544 |
| *Sapelovirus* | Porcine sapelovirus | Po5116 | AY392538 |
| *Sapelovirus* | Avian sapelovirus | TW90A | AY563023.1 |
| *Sapelovirus* | Simian sapelovirus | 2383 | NC_004451.1 |
| *Enterovirus* | Porcine enterovirus B | UKG/410/73 | NC_004441.1 |
| *Teschovirus* | Porcine teschovirus | F65 | NC_003985.1 |
| *Aphtovirus* | Foot-and-mouth disease virus O | OTai | NC_004004.1 |
| *Avihepatovirus* | Duck hepatitis A virus | 03D | NC_008250.1 |
| *Cardiovirus* | Encephalomyocarditis virus | Ruckert | NC_001479.1 |
| *Enterovirus* | Poliovirus | Mahoney | NC_002058.3 |
| *Rhinovirus* | Human rhinovirus | 89 | NC_001617.1 |
| *Erbovirus* | Equine rhinitis B virus | P1436/71 | NC_003983.1 |
| *Hepatovirus* | Hepatitis A virus | HM-175 | NC_001489.1 |
| *Kobuvirus* | Aichi virus | A846/88 | NC_001918.1 |
| *Parechovirus* | Human parechovirus | Gregory | NC_001897.1 |
| *Senecavirus* | Seneca Valley virus | SVV-001 | NC_011349.1 |
| *Tremovirus* | Avian encephalomyelitis virus | Calnek | NC_003990.1 |

**Table S3.** The length of 5’ untranslated region, each part of the open reading frame, 3’ untranslated region and complete genome excepting poly(A) tail.

| Strain | Nucleotide and deduced amino acid lengths of region: | | | | | | | | | | | | | | | |
| --- | --- | --- | --- | --- | --- | --- | --- | --- | --- | --- | --- | --- | --- | --- | --- | --- |
|  | 5’ UTR | L | VP4 | VP2 | VP3 | VP1 | 2A | 2B | 2C | 3A | 3B | 3C | 3D | ORF ^a^ | 3’ UTR | Complete genome |
| KS04105 | 491 | 252  (84)^b^ | 159  (53) | 714  (238) | 702  (234) | 855 (285) | 678  (226) | 315  (105) | 996  (332) | 300  (100) | 66  (22) | 546  (182) | 1386  (462) | 6969  (2323) | 82 | 7542 |
| KS055217 | 491 | 252  (84) | 159  (53) | 714  (238) | 702  (234) | 855 (285) | 678  (226) | 315  (105) | 996  (332) | 300  (100) | 66  (22) | 546  (182) | 1386  (462) | 6969  (2323) | 82 | 7542 |
| KS05151 | 491 | 252  (84) | 159  (53) | 714  (238) | 702  (234) | 879  (293) | 678  (226) | 315  (105) | 996  (332) | 300  (100) | 66  (22) | 546  (182) | 1386  (462) | 6993  (2331) | 82 | 7566 |
| V13 | 443 | 252  (84) | 159  (53) | 714  (238) | 702  (234) | 855 (285) | 678  (226) | 315  (105) | 996  (332) | 300  (100) | 66  (22) | 546  (182) | 1383  (461) | 6966  (2322) | 82 | 7491 |
| csh | 441 | 252  (84) | 159  (53) | 714  (238) | 702  (234) | 879  (293) | 678  (226) | 315  (105) | 996  (332) | 300  (100) | 66  (22) | 546  (182) | 1386  (462) | 6993  (2331) | 68 | 7502 |
| YC2011 | 465 | 252  (84) | 159  (53) | 714  (238) | 702  (234) | 879  (293) | 678  (226) | 315  (105) | 996  (332) | 300  (100) | 66  (22) | 546  (182) | 1386  (462) | 6993  (2331) | 82 | 7540 |

^a^Open reading frame of porcine sapelovirus. ^b^Length of deduced amino acid sequence.

**Table S4.** Comparison of nucleotide and deduced amino acid sequences between the porcine sapelovirus strains.

| Protein | Strain | Percentage sequence identity between PSV strains | | | | | |
| --- | --- | --- | --- | --- | --- | --- | --- |
|  |  | KS04105 | KS055217 | KS05151 | V13 | csh | YC2011 |
| 5’NTR | KS04105 |  |  |  |  |  |  |
|  | KS055217 | 98.9^a^ |  |  |  |  |  |
|  | KS05151 | 99.4 | 99.6 |  |  |  |  |
|  | V13 | 93.2 | 92.8 | 93.2 |  |  |  |
|  | csh | 95.2 | 95.5 | 95.2 | 92.5 |  |  |
|  | YC2011 | 96.1 | 96.3 | 96.1 | 94.3 | 95.2 |  |
| L | KS04105 |  | 94.4^b^ | 100 | 89.3 | 91.7 | 92.5 |
|  | KS055217 | 98.8 |  | 94.4 | 90.9 | 89.3 | 90.1 |
|  | KS05151 | 100 | 98.8 |  | 89.3 | 91.7 | 92.5 |
|  | V13 | 97.6 | 96.4 | 97.6 |  | 89.7 | 88.9 |
|  | csh | 98.8 | 98.8 | 98.8 | 96.4 |  | 92.5 |
|  | YC2011 | 97.6 | 97.6 | 97.6 | 95.2 | 98.8 |  |
| P1 | KS04105 |  | 84.2 | 90.8 | 79.1 | 85.2 | 85.1 |
|  | KS055217 | 93.2 |  | 82.8 | 80.2 | 83.2 | 83.0 |
|  | KS05151 | 97.5 | 92.6 |  | 80.3 | 87.3 | 87.9 |
|  | V13 | 90.0 | 88.4 | 90.2 |  | 79.7 | 79.8 |
|  | csh | 95.7 | 91.7 | 97.3 | 89.8 |  | 89.0 |
|  | YC2011 | 95.9 | 92.5 | 97.7 | 89.8 | 97.5 |  |
| VP4 | KS04105 |  | 86.2 | 96.2 | 78.0 | 80.5 | 84.9 |
|  | KS055217 | 100 |  | 83.6 | 79.2 | 81.1 | 86.2 |
|  | KS05151 | 100 | 100 |  | 78.6 | 81.1 | 86.2 |
|  | V13 | 92.5 | 92.5 | 92.5 |  | 81.8 | 76.7 |
|  | csh | 92.5 | 92.5 | 92.5 | 100 |  | 79.9 |
|  | YC2011 | 100 | 100 | 100 | 92.5 | 92.5 |  |
| VP2 | KS04105 |  | 83.9 | 84.7 | 78.7 | 84.0 | 85.7 |
|  | KS055217 | 95.4 |  | 83.3 | 81.5 | 83.8 | 84.6 |
|  | KS05151 | 97.9 | 95.0 |  | 80.1 | 85.4 | 87.8 |
|  | V13 | 93.7 | 91.2 | 93.7 |  | 79.6 | 80.0 |
|  | csh | 96.2 | 94.5 | 97.1 | 92.0 |  | 88.9 |
|  | YC2011 | 97.1 | 95.4 | 97.9 | 92.9 | 99.2 |  |
| VP3 | KS04105 |  | 83.2 | 88.6 | 80.5 | 86.0 | 84.9 |
|  | KS055217 | 90.2 |  | 83,3 | 81.2 | 83.6 | 82.5 |
|  | KS05151 | 95.7 | 89.7 |  | 82.3 | 92.2 | 92.2 |
|  | V13 | 90.6 | 89.8 | 90.6 |  | 82.2 | 80.6 |
|  | csh | 94.9 | 90.6 | 99.1 | 90.2 |  | 90.7 |
|  | YC2011 | 94.4 | 90.2 | 98.7 | 89.7 | 99.6 |  |
| VP1 | KS04105 |  | 84.8 | 96.7 | 78.4 | 86.4 | 84.9 |
|  | KS055217 | 92.6 |  | 81.8 | 78.6 | 82.7 | 81.6 |
|  | KS05151 | 98.2 | 91.6 |  | 79.1 | 86.0 | 84.9 |
|  | V13 | 86.0 | 86.7 | 86.7 |  | 77.3 | 84.9 |
|  | csh | 96.5 | 90.2 | 96.8 | 85.6 |  | 89.2 |
|  | YC2011 | 95.4 | 90.5 | 96.1 | 86.7 | 95.4 |  |
| P2 | KS04105 |  | 93.4 | 94.0 | 84.9 | 86.6 | 87.4 |
|  | KS055217 | 99.5 |  | 90.0 | 84.9 | 86.8 | 87.8 |
|  | KS05151 | 98.0 | 98.2 |  | 84.8 | 87.1 | 87.5 |
|  | V13 | 94.6 | 94.4 | 94.1 |  | 85.0 | 84.0 |
|  | csh | 97.3 | 97.1 | 97.0 | 94.6 |  | 88.4 |
|  | YC2011 | 97.0 | 97.1 | 96.4 | 94.1 | 97.8 |  |
| P3 | KS04105 |  | 92.5 | 95.0 | 88.1 | 89.7 | 89.9 |
|  | KS055217 | 98.7 |  | 92.5 | 88.1 | 90.3 | 90.3 |
|  | KS05151 | 99.0 | 98.7 |  | 87.8 | 90.2 | 89.9 |
|  | V13 | 96.2 | 96.1 | 96.1 |  | 88.1 | 88.0 |
|  | csh | 98.0 | 97.8 | 98.0 | 95.9 |  | 91.6 |
|  | YC2011 | 98.4 | 98.4 | 98.4 | 96.3 | 98.6 |  |
| 3’ NTR | KS04105 |  |  |  |  |  |  |
|  | KS055217 | 98.8 |  |  |  |  |  |
|  | KS05151 | 100 | 98.8 |  |  |  |  |
|  | V13 | 95.1 | 93.9 | 95.1 |  |  |  |
|  | csh | 95.6 | 97.1 | 92.6 | 92.6 |  |  |
|  | YC2011 | 98.8 | 100 | 98.8 | 93.9 | 97.1 |  |

^a^Lower left of each functional unit indicates the nucleotide sequence identities between the PSV strains.

^b^Upper right of each functional unit indicates the deduced amino acid sequence identities between the PSV strains.
